# Causal necessitarianism and the monotonicity objection

**DOI:** 10.1007/s11229-020-02902-x

**Published:** 2020-10-30

**Authors:** Salim Hirèche

**Affiliations:** Département de philosophie, Université de Genève, Rue de Candolle 2, 1205 Geneva, Switzerland

**Keywords:** Causation, Necessitation, Monotonicity, Blockers, Totality facts, Grounding

## Abstract

Do causes necessitate their effects? Causal necessitarianism (CN) is the view that they do. One major objection—the “monotonicity objection”—runs roughly as follows. For many particular causal relations, we can easily find a possible “blocker”—an additional causal factor that, had it also been there, would have prevented the cause from producing its effect. However—the objection goes on—, if the cause really *necessitated* its effect in the first place, it would have produced it *anyway*—despite the blocker. Thus, CN must be false. Though different from Hume’s famous attacks against CN, the monotonicity objection is no less important. In one form or another, it has actually been invoked by various opponents to CN, past and present. And indeed, its intuitive appeal is quite powerful. Yet, this paper argues that, once carefully analysed, the objection can be resisted—and should be. First, I show how its success depends on three implicit assumptions concerning, respectively, the notion of cause, the composition of causal factors, and the relation of necessitation. Second, I present general motivations for rejecting at least one of those assumptions: appropriate variants of them threaten views that even opponents to CN would want to preserve—in particular, the popular thesis of *grounding* necessitarianism. Finally, I argue that the assumption we should reject is the one concerning how causes should be understood: causes, I suggest, include an element of completeness that excludes blockers. In particular, I propose a way of understanding causal completeness that avoids common difficulties.

## Introduction

An important philosophical question about causation concerns its modal status: Do causes necessitate their effects? Causal necessitarianism (CN) is the view that they do. Proponents of CN can be found throughout the history of philosophy—from Aristotle to some contemporary “dispositional essentialists”.^1^ CN has also been the target of various attacks, most famously from Hume.^2^ This paper focuses on one particular objection: the “monotonicity objection”, which runs roughly as follows. For many particular causal relations, it seems that we can find a possible “interferer”, or “blocker”—an additional causal factor that, had it also been there, would have prevented the cause from producing its effect. However—the objection goes on—, if the cause really *necessitated* its effect in the first place, it would have produced it *anyway*—even with the blocker. Therefore, CN is false.

Though different from Hume’s attacks,^3^ the monotonicity objection is no less important—in particular, its intuitive appeal is quite powerful. Indeed, it has been invoked, in one form or another, by various opponents to CN, past and present.^4^ Yet, it has not, I think, received the attention that it deserves: more needs to be said about its precise formulation, its background assumptions, and the possible strategies to answer it. This is what I aim to do in this paper.

First, I offer an analysis of the objection. I propose a formulation of it as a valid argument, consisting of three premises which may be briefly stated as follows: some causes have possible blockers; necessitation is monotonic; necessitation is without exceptions. And I argue that those premises, in turn, rely on three implicit assumptions: causal factors compose classically, namely non-holistically (when they obtain together, each of them still individually obtains); causes do not include any completeness component (e.g. a no-blocker or totality fact); necessitation is to be understood classically, namely as a standard strict conditional. This analysis sheds light on the main strategies to undermine the argument, each involving the rejection of one assumption.

Second, I present general motivations for rejecting at least one of those assumptions: I argue that appropriate variants of them threaten views that even opponents to CN would want to preserve: the modally weaker view that causes *nomically* necessitate their effects; and the quite popular view that *grounds* necessitate what they ground. Finally, I argue that the assumption that should be rejected, both as the best way to undermine the argument and for independent reasons, is the one concerning the content of causes: causes, I argue, do include a completeness component that excludes blockers. In particular, I propose a way of understanding this completeness component that avoids common drawbacks (e.g. making causal necessitation trivial, or vacuous).

## Analysing the objection

### Example and preliminaries

Before formulating the objection more precisely, let us consider an example used by two of its main contemporary proponents, Anjum and Mumford (2011b, pp. 59–69). I will not go through all the details, but focus on what I take to be most relevant to our main purpose: understand how the reasoning behind the objection works, and put some useful preliminaries in place. Suppose that, in a given room, the temperature evolves in a certain way—say, it rises at a certain rate. We are interested in what causes that particular rise in temperature. The natural focus will be on relevant causal factors, namely things that are causally relevant to the evolution of temperature in that room—e.g. a heater’s being on in a corner of the room, a window’s being open, some people’s being present, and so on –, as opposed to things that are not—e.g. the fact that the day is Tuesday.^5^

In general, we will assume that, for each particular effect, we can objectively identify the relevant causal factors—e.g. the causal factors that are relevant to the temperature in a room, when we are considering what causes a particular evolution of temperature in that room; or relevant to the movement of a given object, when we are considering what causes that object to move in a particular way. This should not be taken to imply the epistemic claim that relevant causal factors are determinable *in practise*—that we can establish an explicit list of them. In many actual cases, this would be very complicated, and it would require an extensive knowledge of many different facts, laws of nature, and so on. Rather, the intended assumption is a metaphysical one: for each particular effect, as a matter of fact, certain things, but not others, are relevant causal factors, so that it makes sense to refer to them. This only implies that they are determinable *in principle*. This assumption seems reasonable—and, importantly, it is equally acceptable to both sides in the debate.

Unless otherwise specified, “cause”, in what follows, will always mean *full* cause, as opposed to partial cause; a cause will be assumed to include all the various relevant causal factors, not only some of them. First, although we often talk, for convenience, as if the relevant causal factors could be divided into “the cause” and “the background or surrounding conditions”, I take it that the division is largely perspectival, rather than properly metaphysical: it mainly amounts to giving a special status to relevant causal factors that are particularly salient or interesting to us in a given context.^6^ From a metaphysical point of view, it is all the different relevant causal factors together that bring about the effect. Second, if by “cause” we meant partial cause—only part of the relevant causal factors –, a discussion of CN would be of little interest anyway—the thesis would be trivially false. In our example, the effect is a particular rise in temperature in a room—call that effect “C”. The cause, accordingly, will include the plurality A_1_, A_2_, … of all the causal factors relevant for the evolution of temperature in that room: the heater’s being on, the window’s being open, and so on. For simplicity, I will call “A” the plurality A_1_, A_2_, … of relevant causal factors.

Thus, we are assuming that A caused C. But did A *necessitate* C? Here is a reason to think that it did not. It seems clear that there could have been a further relevant causal factor B—say, a powerful cooler that would have been on at the same time—such that A and B together would have caused the temperature in the room to *decrease* instead. Thus, we would have had A and B, but not C. B provides an example of what Mumford and Anjum call a possible “additive interference”—and that I will call a possible “blocker”, as it would have prevented C from being caused. Now, how does *this* show that A did not necessitate C in the first place? The idea is that, if A really did necessitate C, then it would have done so in any situation, including one where B is also there. Necessitation, in other words, seems to be subject to a principle of “antecedent strengthening”, or “monotonicity”. As Mumford and Anjum (2011b, p. 68) put it:

What we have, therefore is an antecedent strengthening argument against the necessitation of an effect by its cause. If C necessitated E, then C, plus anything added, should also produce E.

Or, as Markus Schrenk (2010, p. 731) puts it,

The crucial point is that necessity is *monotonic*: if C *necessarily* leads to E, so must C plus the antidote A.

According to this line of reasoning, the fact that a cause actually succeeded in causing an effect is irrelevant: even if no blocker actually intervened, the point is that one *could* have. Moreover, the fact that we are considering, not a single relevant causal factor, but all the relevant causal factors—together constituting what we may call the *resultant* causal factor—is also irrelevant:

[W]e can see that this antecedent strengthening consideration that counts against the necessitation of its manifestation by a power applies just as much to any resultant power as it does to the individual component powers. The resultant too *only disposes towards* an outcome for it too is subject to the possibility of additive interference. (Mumford and Anjum 2011b, p. 69; emphasis added.)

### The monotonicity argument against causal necessitarianism

The above gave us an illustration of how the reasoning works: starting from potential blockers, we get a failure of monotonicity, which in turn leads to a failure of necessitation. This may be formulated as a general argument against CN:

(Monotonicity Argument Against CN)

**(POSSIBLE BLOCKERS) Some causes have possible blockers**: For some A and C, A causes C, but it is possible that there is a further relevant causal factor B, A and B obtain together, and C does not obtain.

**(MONOTONIC NECESSITATION) Necessitation is monotonic**: For any A and C, if A necessitates C, then, for any B, A and B together necessitate C.

**(EXCEPTIONLESS NECESSITATION) Necessitation is exceptionless**: For any A and C, if A necessitates C, then it is not possible that A obtains but C does not.

Therefore,

CN is false: It is not the case that, for any A and C, if A causes C, then A necessitates C.

Note, first, that I take A, B and C to be broad pluralities of facts—i.e. one or more facts. Typically, the effect C is a single fact, whereas the cause A is a strict plurality of facts—in which case “A obtains” is short for “A_1_, A_2_, … obtain together”. However, nothing crucial, in the following discussion, should rely on understanding causation specifically in terms of facts (as opposed to other fact-like entities, such as events).^7^ This choice is mainly practical: in particular, at some points, I will establish parallels between causation and grounding, and a common way of understanding the latter is precisely as a relation between facts.^8^ Following usual conventions, I will sometimes use “[p]” as short for “the fact that p”, and “<p>” for “the proposition that p”. Second, for the main proponents of the monotonicity objection, blockers are possible for *all* causes.^9^ Thus, in a more accurate reconstruction, premise (POSSIBLE BLOCKERS) would be strengthened—as (POSSIBLE BLOCKERS *FOR ALL CAUSES*) –, and the resulting argument would threaten, not only CN, but *partial* CN—the view that *some* causes necessitate their effects. For the time being, I will consider the weaker argument formulated above, as it suffices as an argument against CN.

Third, the above argument is clearly valid. By (POSSIBLE BLOCKERS), for some A and C, A causes C, but there could have been a blocker, namely a further relevant causal factor B such that A and B obtain together, and C does not. In particular, given (EXCEPTIONLESS NECESSITATION), there is a B such that A and B together do not necessitate C. This implies, given (MONOTONIC NECESSITATION), that A does not necessitate C. As we assumed that A causes C, CN must be false. Moreover, all three premises are at least *prima facie* plausible. As the room temperature example illustrated, (POSSIBLE BLOCKERS) is based on the apparently obvious fact that, at least for many particular causal relations, there could have been a blocker. As regards (MONOTONIC NECESSITATION), it is quite plausible as well: if A necessitates C, it seems that it should have done so in whatever alternative circumstances—in particular, together with any other B. Finally, as regards (EXCEPTIONLESS NECESSITATION), A necessitating C sounds trivially incompatible with the possibility that A obtains without C also obtaining.

### Three implicit assumptions

It may indeed be tempting to conclude that (POSSIBLE BLOCKERS), (MONOTONIC NECESSITATION) and (EXCEPTIONLESS NECESSITATION) are, not only *prima facie* plausible, but quite clearly true. And the main reason, I suggest, is that they implicitly rely on three further assumptions that are usually just taken for granted:

**(Classical Composition) The composition of relevant causal factors is classical**: when a plurality of relevant causal factors A = A_1_, A_2_, … obtain together, each of them still fully obtains—i.e. “A_1_, A_2_, … obtain together”, or for short “A obtains”, is correctly formalised as a classical conjunction: <A_1_ obtains> ˄ <A_2_ obtains> ˄ ….

**(No Completeness) A cause (i.e. a full cause) includes the relevant causal factors, but no completeness fact** (e.g. a “totality” fact, a “*ceteris paribus*” fact, a “no-blocker” fact).

**(Classical Necessitation) A necessitates C iff □(<A obtains>  →  <C obtains>)**, where → is the material conditional and □ is the necessity operator with the usual possible-worlds semantics (PWS).

These assumptions are quite natural and may seem to need no justification. As regards (Classical Composition), in the room temperature example, there is a plurality A = A_1_, A_2_, … of different causal factors (the fact that the heater is on, the fact that the window is open, and so on). Intuitively, the composition of A_1_, A_2_, … is classical: when they obtain together, each of them still fully obtains; accordingly, although this may simply be formalised, for simplicity, as one proposition— <A obtains> , or <A_1_, A_2_, … obtain together> –, it may also be correctly formalised as a classical conjunction: <A_1_ obtains> ∧ <A_2_ obtains> ∧…. Likewise, to describe the counterfactual situation where a further relevant causal factor B (the powerful cooler) obtains, we may simply add it as a further conjunct: <A_1_ obtains> ∧ <A_2_ obtains> ∧… ∧ <B obtains> —or, for short, <A obtains> ∧ <B obtains> . And similar remarks would apply if, for instance, the effect were the acceleration of an object *o*, and the relevant causal factors, A = A_1_, A_2_, …, were the forces being exerted on it: A_1_ = [Force f_1_ is exerted on *o*], A_2_ = [Force f_2_ is exerted on *o*], …

As regards (No Completeness), completeness facts (*ceteris paribus* facts, totality facts, and the like) are often regarded as unwanted for various reasons. For one thing, including a completeness fact in the cause simply seems superfluous—even if “cause”, as it is assumed here, means *full* cause, all you need to get a cause is all the relevant causal factors, taken together. Moreover, it may seem to make the claim that the cause is necessitating circular, or vacuous—something roughly amounting to the claim that the cause, *excluding* possible cases where it would have failed to produce its effect, would have produced it in all possible cases. A third kind of reason concerns the formulation of the completeness fact itself; one may think that it is doomed to be too complex or insufficiently clear. Finally, as regards (Classical Necessitation), using the strict conditional with the usual PWS is a very common and simple way of formalising the relation of necessitation—indeed, one that yields, in at least most cases, results that fit our intuitions.

Later on, I will argue that each assumption (or something close to it) is plausibly needed for the monotonicity argument to work—that if we drop one of them, there is a clear way to undermine the argument. Now, let us see more precisely how they jointly make it work. Consider first premise (POSSIBLE BLOCKERS). As we have seen, for at least many causal relations, it seems that we can find a possible blocker. The most natural way to avoid that possibility would be to say that the cause, in addition to relevant causal factors, includes some appropriate completeness fact excluding the blocker; but this is precisely what (No Completeness) does not allow. Thus, given (No Completeness), it seems that (POSSIBLE BLOCKERS) should be accepted. Premise (MONOTONIC NECESSITATION) should also be accepted, given (Classical Composition) and (Classical Necessitation). By (Classical Necessitation), A necessitates C just in case □(<A obtains>  →  <C obtains>) holds. In particular, if □(<A obtains>  →  <C obtains>) holds, then □((<A obtains> ˄ <B obtains>) →  <C obtains>) holds for any B: for any possible world W, if both A obtains and B obtains in W, then A obtains in W; and as by hypothesis C obtains in any world where A obtains, C also obtains in W. This clearly establishes the monotonicity of necessitation for all cases where A and B compose classically. Are there cases where they do not? In the present context, relevant cases would be ones where A and B are, more precisely, relevant causal factors composing non-classically; but this is precisely what (Classical Composition) excludes. Hence, we have no reason, given (Classical Composition) and (Classical Necessitation), to reject (MONOTONIC NECESSITATION). Finally, given (Classical Necessitation), A necessitates C just in case □(<A obtains>  →  <C obtains>) holds, namely just in case it is not possible that A obtains without C also obtaining (there is no possible world in which A obtains and C does not). Hence, (EXCEPTIONLESS NECESSITATION) should also be accepted.

## Addressing the objection

### General motivations: causal regularity lost

Apparently, there are good reasons to keep (Classical Composition), (No Completeness) and (Classical Necessitation); but if we do, the monotonicity argument against CN quite clearly works. Some may not see this as an important cost: after all, unless you hold some necessitarian view about causal laws in the first place,^10^ it is unlikely that you will even consider CN as plausible. However, the costs of keeping those three assumptions are not limited to the loss of CN. First, it most plausibly leads to the falsity of causal *nomic* necessitarianism (CNN): the weaker view that, if A causes C, then A *nomically* necessitates C—where nomic necessitation is necessitation *given* the laws of nature (in particular, the causal ones). As I noted earlier, some (e.g. dispositional essentialists) understand laws of nature as universal generalizations that are metaphysically necessary; on their view, CNN is just as strong as CN. However, many rather take laws to have a primitive modal force, strictly weaker than metaphysical necessity.^11^ Indeed, on broadly Humean accounts, laws are contingent: they are simply true universal generalizations, perhaps meeting some additional (epistemic or practical) criteria.^12^ On such non-necessitarian views of laws, CNN is clearly weaker than CN. Yet, CNN, whatever modal status we attribute to laws, faces an analogous monotonicity argument:

(Monotonicity Argument against Causal Nomic Necessitarianism)

**(NOMICALLY POSSIBLE BLOCKERS) Some causes have nomically possible blockers**: For some A and C, A causes C, but it is nomically possible that there is a further relevant causal factor B, A and B obtain together, and C does not obtain.

**(MONOTONIC NOMIC NECESSITATION) Nomic necessitation is monotonic**: For any A and C, if A nomically necessitates C, then, for any B, A and B together nomically necessitate C.

**(EXCEPTIONLESS NOMIC NECESSITATION) Nomic necessitation is exceptionless**: For any A and C, if A nomically necessitates C, it is nomically impossible that A obtains, and C does not.

Therefore,

CNN is false.

The argument is valid, and its premises quite clearly follow from assumptions (Classical Composition) and (No Completeness), and an adapted version of (Classical Necessitation):

(Classical Nomic Necessitation) A nomically necessitates C *iff *□_N_(<A obtains>  →  <C obtains>), where □_N_ is the nomic necessity operator with the usual PWS (with possible worlds restricted to nomically possible worlds).

Just like (POSSIBLE BLOCKERS) in the original argument, (NOMICALLY POSSIBLE BLOCKERS) should be accepted, given (No Completeness). The key point is that, usually, when considering a particular causal relation, and looking for a possible blocker, we do not even need to think about a situation obtaining in a distant metaphysically possible world—one with different causal laws, or alien kinds. Clearly, for at least many causes, we will find a *nomically possible* world where A obtains together with a further relevant causal factor B, and C does not obtain—for instance, the powerful cooler, in the above temperature case, or the additional force, in the above acceleration case, were not only possible, but *nomically possible* blockers. As regards premises (MONOTONIC NOMIC NECESSITATION) and (EXCEPTIONLESS NOMIC NECESSITATION), they follow, by the same reasoning as before, from (Classical Composition) and (Classical Nomic Necessitation).

I take it that anyone accepting (Classical Necessitation) would also accept (Classical Nomic Necessitation): the reasons one may have for understanding necessitation *tout court* or nomic necessitation in the classical way should essentially be the same. If so, then what seems to be the best way to make the monotonicity argument against CN work—i.e. to assume (Classical Composition), (No Completeness) and (Classical Necessitation)—quite naturally leads to the loss of CNN. To put it otherwise, what is ultimately at stake with the monotonicity objection is not the idea that causal relations involve metaphysically necessary connections, but the more general idea that they involve corresponding lawful regularities—non-accidental “constant conjunctions”. And this idea may be equally appealing to necessitarians and non-necessitarians about laws.

### General motivations: grounding necessitarianism threatened

Some may remain unimpressed by the above considerations, thinking that causation is a particularly “fuzzy” matter anyway and that, although casual talk of causal regularity (“same cause, same effect”) is quite common, there is no such thing, strictly speaking.^13^ However, the consequences of keeping (Classical Composition), (No Completeness) and (Classical Necessitation) may not be limited to the realm of causation: it may ultimately affect another sort of explanatory relation, which has become central in recent philosophical debates—metaphysical grounding.^14^ Even among opponents to CN, many would probably be keen on preserving grounding necessitarianism (GN): the view that, if A grounds (i.e. fully grounds) C, then A necessitates C. This position might seem natural: grounding, often considered as a “stronger” explanatory relation—*metaphysical* explanation as opposed to “mere” causal or nomic or natural explanation –, should yield necessitation, even though causation does not. Indeed, it seems fair to say that, in the grounding literature, GN is majority.^15^ And sometimes this modal feature is precisely presented as one of the main ways in which grounding differs from causation.^16^ Yet, GN is threatened by an analogous argument:

(Monotonicity Argument against Grounding Necessitarianism)

**(POSSIBLE BLOCKERS FOR GROUNDING) Some grounds have possible blockers**: For some A and C, A grounds C, but it is possible that there is a further relevant grounding factor B, A and B obtain together, and C does not obtain.

**(MONOTONIC NECESSITATION) Necessitation is monotonic**: For any A and C, if A necessitates C, then, for any B, A and B together necessitate C.

**(EXCEPTIONLESS NECESSITATION) Necessitation is exceptionless**: For any A and C, if A necessitates C, then it is not possible that A obtains but C does not.

Therefore,

GN is false.

The premises of this valid argument quite clearly follow from assumption (Classical Necessitation), together with grounding versions of (Classical Composition) and (No Completeness):

(Classical Composition for grounding) The composition of relevant grounding factors is classical: when a plurality of relevant grounding factors A = A_1_, A_2_, … obtain together, each of them still fully obtains—i.e. “A_1_, A_2_, … obtain together”, or for short “A obtains”, is correctly formalised as a classical conjunction: <A_1_ obtains> ˄ <A_2_ obtains> ˄ ….

(No Completeness for grounding) A ground (i.e. a full ground) includes the relevant grounding factors, but no completeness fact (e.g. a “totality” fact, a “ceteris paribus” fact, a “no-blocker” fact).

Premise (EXCEPTIONLESS NECESSITATION) follows from (Classical Necessitation), as before. Assuming also (Classical Composition for grounding), it seems that we have no reason, in this context, to deny (MONOTONIC NECESSITATION). Now consider premise (POSSIBLE BLOCKERS FOR GROUNDING). First, whereas it seems plausible that, assuming (No Completeness), all causes have possible blockers, it seems much less plausible that all grounds do, even assuming (No Completeness for grounding). For instance, let A be [Chris is an unmarried man], and let C be [Chris is a bachelor]. Arguably, A grounds C—with A including no sort of completeness fact. However, we would not be able to find any fact B such that A and B could have obtained together, without C obtaining. It seems that this ground (and you will easily find other examples) has no possible blocker.

Yet, this should not lead us to the extreme conclusion that blockers are impossible in all grounding cases. Indeed, some are very similar to causal cases in this respect. Let C be the following restricted accidental generalization: [∀x(Mx → Hx)], where M and H are the property of being a mammal in Sarah’s garden, and the property of being a hedgehog, respectively. And suppose that C obtains: the mammals in Sarah’s garden are indeed three hedgehogs—*a*, *b* and *c*. For some philosophers, there are good reasons to think that the right ground for C is simply the plurality of relevant grounding factors constituted by C’s instances—i.e. A = [Ha], [Hb], [Hc].^17^ The main other candidate would be A^+^, namely A *plus* a totality fact T, with T = [*a*, *b* and *c* are the only M’s].^18^ However, as a sort of completeness fact, T cannot be part of the ground if we assume (No Completeness for grounding). This leaves us with A grounding C. Clearly, our ground has possible blockers—e.g. B, the fact that there is a fox in Sarah’s garden.

As a different illustration, suppose that, in my room, there is a collection of different objects put together, with certain shapes, arranged in a certain way, such that the whole thing forms a complex object, *o*, that has the property of being a chair—suppose that there is a reasonable functional definition of a chair, including the condition that someone may actually be sitting on it. Arguably, A, [Object *o* is in my room], grounds C, [There is a chair in my room]. But now consider a further relevant grounding factor that could have obtained: B, the fact that there is an additional leg attached to the original object, *o*, and that this leg is much longer than the other four ones, making *o* lose its stability. Call *o’* the resulting object—*o’* is just *o plus* the additional leg, so that *o* would still be in my room if *o’* were. Suppose that *o’* would not have the property of being a chair—one could not be sitting on it anymore. So A grounds C, but A *could* have obtained together with B, without C obtaining. One may be tempted to say that A should include some sort of completeness fact excluding B; but this is not an available option if we assume (No completeness for grounding).

As a last example, suppose that I like the taste of chocolate very much: when I ate some yesterday, the taste was pleasant—as it always is. We may say my tasting chocolate (perhaps with further facts about my tasting preferences)—fact A—was the ground for my having a pleasant tasting experience—fact C. Now, instead of simply tasting chocolate, I *could* have tasted both chocolate *and* fish at the same time. B, the fact that I taste fish, would have been a further relevant grounding factor for my tasting experience, and it would have obtained together with A. In this alternative situation, C might well have failed to obtain—my tasting experience might not have been so pleasant.

Given such grounding examples,^19^ it seems that (POSSIBLE BLOCKERS FOR GROUNDING) is true if we assume (No Completeness for grounding); and assuming also (Classical Composition for grounding) and (Classical Necessitation), (MONOTONIC NECESSITATION) and (EXCEPTIONLESS NECESSITATION) are true as well—which leads to the falsity of GN. Earlier, I assumed that anyone accepting (Classical Necessitation) would most plausibly accept (Classical Nomic Necessitation) as well. Similarly, I suggest that (Classical Composition for grounding) should probably be accepted if (Classical Composition) is: if the composition of relevant causal factors is always classical, the same should be true of relevant grounding factors. It is far from clear why one would think that the composition of A and B is classical when, for instance, A and B correspond to different forces being exerted on a given object, or to different causal factors relevant for the temperature in a room, but *not* when, for instance, A and B correspond to different mammals being in Sarah’s garden. Likewise, I suggest that (No Completeness for grounding) should be accepted if (No Completeness) is: if no cause includes any completeness fact, then presumably the same goes for grounds. The idea is that what is at stake is the more general question whether we should accept completeness facts in explanatory facts, be they causal or metaphysical, and that the reasons for accepting them in one case or the other should essentially be the same (more on this in Sect. 5.1). The above examples already provide some motivation for this claim: it is unclear why one would accept a completeness fact in the ground when what is to be grounded is the fact that all mammals in Sarah’s garden are hedgehogs, or that there is a chair in my room, but exclude any completeness fact from the cause when, for instance, what is to be caused is the acceleration of an object *o*. Indeed, the analogy between those cases seems quite clear.

The discussion in later sections will bring some further motivation for the claims that (Classical Composition for grounding) should be accepted if (Classical Composition) is, and that (No Completeness for grounding) should be accepted if (No Completeness) is. Assuming them for now, we can conclude that what seems to be the best way to make the monotonicity argument against CN work—namely holding (Classical Composition), (No Completeness) and (Classical Necessitation)—would lead, in addition, to the loss of GN. Of course, opponents to GN may remain unimpressed by such considerations. They may even take them as further support for their own view—in general, it is not uncommon for opponents to GN to assume the falsity of CN, and to rely on such analogies between causation and grounding to motivate their position.^20^ On the other hand, many philosophers endorse GN. For them, if the above considerations are sound, abandoning at least one assumption among (Classical Composition), (No Completeness) and (Classical Necessitation) should be an attractive option.^21^

### Specific strategies: non-exceptionless necessitation, non-monotonic necessitation, holistic composition, completeness

I have presented general reasons why one may want to abandon at least one assumption among (Classical Composition), (No Completeness) and (Classical Necessitation). But which one(s), if any, should be abandoned, and on what independent grounds? There are four basic strategies to resist the monotonicity argument against CN, each one involving the rejection of one assumption. Those strategies are independent from each other—pursuing one of them successfully is sufficient to undermine the argument, but it is compatible with pursuing any other strategy simultaneously. First, one may reject (Classical Necessitation), arguing that necessitation is not exceptionless—or perhaps that the notion of necessitation which is relevant in the context of causation is not. This would clearly undermine premise (EXCEPTIONLESS NECESSITATION). In what follows, I will not consider this strategy as an option: CN, as I understand it here, involves the unqualified notion of necessitation, namely necessitation *tout court*.^22^ And I will assume that this notion would not deserve the name if it were not strong enough to exclude exceptions.

Second, one may argue that (Classical Necessitation) should be replaced, not (necessarily) with a non-exceptionless formalisation, but with a non-monotonic one—thereby undermining (MONOTONIC NECESSITATION). This strategy (or something similar) has actually been defended,^23^ but I will not pursue it here. There may indeed be independent reasons to go for an alternative, non-monotonic formalisation of necessitation that is *stronger* than (Classical Necessitation)—say, (Stronger Non-Monotonic Necessitation), a formalisation that is non-monotonic because it requires, *in addition* to what (Classical Necessitation) requires, that the antecedent be relevant to the consequent.^24^ However, although (Stronger Non-Monotonic Necessitation) would indeed undermine (MONOTONIC NECESSITATION) as it stands, it seems that the proponent of the monotonicity argument would have an easy response. She could replace (MONOTONIC NECESSITATION) by the following premise:

(ALTERNATIVE MONOTONICITY) For any A and C, if A necessitates C, then, for any B, it is not possible (i.e. classically possible) that A and B obtain together, and C does not obtain.

Still assuming (Classical Composition), (ALTERNATIVE MONOTONICITY) is as plausible with (Stronger Non-Monotonic Necessitation) as it is with (Classical Necessitation). And now (POSSIBLE BLOCKERS) and (ALTERNATIVE MONOTONICITY) suffice as the two premises of a new valid argument against CN—indeed a successful one, given (No Completeness). More generally, as long as we assume (Classical Composition), (ALTERNATIVE MONOTONICITY) would hold, not only assuming (Stronger Non-Monotonic Necessitation), but *any* formalisation that is stronger than (Classical Necessitation). Thus, if we wanted to save CN just by replacing (Classical Necessitation) with an alternative formalisation—while keeping (No Completeness) and (Classical Composition) –, the alternative formalisation would have to be weaker—such that, *even* assuming (Classical Composition), (ALTERNATIVE MONOTONICITY) would be false. However, it seems that such a formalisation would then be too weak to yield an intuitive notion of necessitation. The strategy under consideration, then, seems to face a sort of dilemma. Be that as it may—for the rest of this paper, I will assume that necessitation is correctly formalised with (Classical Necessitation).

The third strategy consists in undermining (MONOTONIC NECESSITATION) by rejecting, not (Classical Necessitation), but (Classical Composition), claiming that the way relevant causal factors compose is not (always) classical. Something like this strategy has also been at the centre of a debate between critics and proponents of the monotonicity objection.^25^ As it may indeed look more promising than the first two ones, I will briefly discuss it in the next part of this paper (Sect. 4). Finally, the fourth strategy is to reject (No Completeness), arguing that causes *do* include some sort of completeness fact excluding blockers. This would be a way to undermine (POSSIBLE BLOCKERS)—causes would not have possible blockers, strictly speaking. This strategy will also be discussed, indeed defended, in some more detail (Sect. 5).

It is worth noting that those two strategies, though distinct, are similar in that both aim at arguing that, in the alleged blocker situations described, the cause would not obtain. Yet, as we will see in more detail, the two strategies differ as to why exactly it is so. On the former strategy, the obtaining of the cause together with the blocker, though possible, would amount to the obtaining of a holistic fact, in which the cause would somehow be lost—it would no longer obtain (in the way it actually does). By contrast, on the latter strategy, the obtaining of the cause together with the blocker is simply impossible, as they are incompatible.

## Against the holistic composition strategy

### Holistic composition, monotonicity, and blockers

Is it so clear that causal factors compose classically? Perhaps the various relevant causal factors A_1_, A_2_, … for the evolution of temperature in a given room form a complex web of interdependent factors, whose obtaining together is best understood as the obtaining of one holistic fact, H—not reducible to the obtaining of A_1_, the obtaining of A_2_, etc. Likewise, if a *further* relevant causal factor B (a powerful cooler’s being on) had obtained together with H, the latter would no longer have obtained (as it would have without B): the obtaining of H together with B, though possible, would have amounted to the obtaining of a new holistic fact, H*—in which neither H nor B, the two “ingredients” that would have holistically composed into H*, would have been preserved.^26^

Such considerations may motivate the rejection of (Classical Composition). Indeed, simply considering that (Classical Composition) *might* be false should naturally lead us to reject (MONOTONIC NECESSITATION) in its current form. When A necessitates C, it may be intuitive that A and B together should also necessitate C, but *only if* the obtaining of A and B together still implies the obtaining of A—*not* if it amounts to the obtaining of a holistic fact instead. (MONOTONIC NECESSITATION) may then be replaced with a weaker, more plausible premise:

(CLASSICALLY MONOTONIC NECESSITATION) Necessitation is *classically* monotonic: For any A and C, if A necessitates C, then, for any B *such that A and B compose classically,* A and B together necessitate C.

Accordingly, (POSSIBLE BLOCKERS) should be replaced with a premise requiring that the possible blockers be such that they would have composed *classically* with the cause. The resulting new valid argument is the following:

(*Classical Monotonicity* Argument against CN)

**(POSSIBLE CLASSICAL BLOCKERS) Some causes have possible classical blockers**: For some A and C, A causes C, but it is possible that there is a further relevant causal factor B, *A and B compose classically*, A and B obtain together, and C does not obtain.

**(CLASSICALLY MONOTONIC NECESSITATION) Necessitation is classically monotonic**: For any A and C, if A necessitates C, then, for any B *such that A and B compose classically,* A and B together necessitate C.

**(EXCEPTIONLESS NECESSITATION) Necessitation is exceptionless**: For any A and C, if A necessitates C, then it is not possible that A obtains but C does not.

Therefore,

CN is false.^27^

With this modified argument, the dialectics of the discussion changes. Unlike (MONOTONIC NECESSITATION), (CLASSICALLY MONOTONIC NECESSITATION) does not depend on (Classical Composition) anymore: it simply follows from (Classical Necessitation), just as (EXCEPTIONLESS NECESSITATION) does. What does rely on (Classical Composition), as well as on (No Completeness), is now (POSSIBLE CLASSICAL BLOCKERS). For whether causes have possible *classical* blockers depends on two things. First, it depends on whether causes have *any* sort of possible blockers in the first place. As we have seen, assuming (No Completeness), many causes, plausibly all of them, quite clearly do. The second question, then, is whether those possible blockers are classical; and this crucially depends on (Classical Composition), and more generally on whether all, some or no pluralities of causal factors compose classically.

### Can holistic composition save necessitarianism?

In the new dialectical situation just described, one may simply reject (Classical Composition), arguing for holistic composition—while keeping (Classical Necessitation) and (No Completeness). In principle, pursuing this strategy successfully would be sufficient to undermine (POSSIBLE CLASSICAL BLOCKERS). But can it be pursued successfully? It is difficult to deny that at least *some* causes have possible classical blockers. Suppose that A includes the forces exerted on an object *o*, and that A causes C, the fact that *o* accelerates accordingly. B, the fact that a *further* force is exerted on *o*, is quite clearly a possible blocker. And there seems to be no reason to think that A and B would not have composed classically: intuitively, there is nothing in the additional force’s being exerted on *o* that would have prevented the initial set of forces from being fully exerted on *o*—and vice versa.^28^

Some have argued that composition may be classical in this and similar cases, but holistic in other cases: roughly, composition is classical when it can be described as “linear”, as in the case of Newtonian forces, whose composition can simply be formalised as an addition of vectors; and it is holistic when composition is more complex than simple addition.^29^ However, even assuming that we can draw such a distinction between “linear” and “non-linear” composition, it is not even clear that composition should be holistic in the latter case. For instance, in the room temperature example, a precise mathematical description of how the various relevant causal factors combine to bring about the effect may involve quite complex, non-linear equations; and their composition may, in that sense, be considered as “non-linear”. Yet, there may still be a legitimate impression that the actual relevant causal factors (the heater’s being on, the window’s being open, etc.) compose classically with each other, and would have composed classically with a further relevant causal factor (the powerful cooler).

Be that as it may, even if composition were holistic for *some* types of relevant causal factors, it would not suffice to falsify (POSSIBLE CLASSICAL BLOCKERS). Indeed, it may not even suffice to falsify the stronger premise, (POSSIBLE CLASSICAL BLOCKERS *FOR ALL CAUSES*), and undermine the corresponding classical monotonicity argument against *partial* CN, based on (POSSIBLE CLASSICAL BLOCKERS FOR ALL CAUSES), (CLASSICALLY MONOTONIC NECESSITATION) and (EXCEPTIONLESS NECESSITATION). For unless some causes have no possible blocker whatsoever—which is quite implausible, given (No Completeness) –, undermining (POSSIBLE CLASSICAL BLOCKERS FOR ALL CAUSES) requires showing that composition would have been holistic, not simply for some causes and *some* of their possible blockers, but *any* of their possible blockers—which looks like a significantly more difficult task. In sum, it is unclear whether the holistic composition strategy can ultimately save partial CN; and it seems indeed very unlikely that it can save CN itself.

Note that, in this respect, the grounding case is analogous. Based on some putative examples of holistic composition of grounding factors (e.g. my tasting chocolate and my tasting fish), one may reject (Classical composition for grounding) and replace the initial monotonicity argument against GN with a *classical monotonicity* argument. However, it is very unlikely that there are enough cases of holistic composition to undermine this argument: composition is clearly classical for at least many types of grounding factors—e.g. the mammals being in Sarah’s garden. Thus, it seems that the causal case and the grounding case are on par with respect to the holistic composition strategy: it is plausibly doomed to fail in both cases.

## In defence of the completeness strategy

### Motivating completeness

The fourth strategy is to reject (No Completeness), arguing that causes *do* include some appropriate completeness fact excluding possible blockers—and saying more about what form this completeness fact would take, a question that I will address shortly (Sects. 5.2–5.3). Thus, the cause is not simply A, namely the relevant causal factors, but A^+^, namely A *plus* the completeness fact. One motivation for pursuing this strategy concerns the specific purpose of saving, not just partial CN, but CN itself, from the monotonicity objection. For this requires a strategy that is applicable to *all* causation—to *any* cause (that has at least a putative possible blocker) and *any* putative possible blocker. The strategy discussed above was not general in that sense. The idea was not to say that the obtaining of the cause together with the alleged blocker was impossible, but that it amounted to the obtaining of a holistic fact, in which the cause was not preserved. Thus, the strategy was only applicable to cases involving a specific sort of causal factors, and a resulting specific mode of composition. By contrast, the completeness strategy, if applicable at all, is applicable to all causes, however the causal factors involved compose: the obtaining of the cause together with a blocker is simply impossible, because any putative blocker, classical or holistic, is incompatible with the completeness fact. Thus, it offers a more promising way to undermine, not just (POSSIBLE CLASSICAL BLOCKERS FOR ALL CAUSES), but (POSSIBLE CLASSICAL BLOCKERS), and indeed (POSSIBLE BLOCKERS). (And of course, if (POSSIBLE BLOCKERS) is false, so is, *a fortiori*, (NOMICALLY POSSIBLE BLOCKERS) in the argument against CNN.)

Beyond such “strategic” motivations for completeness, what about independent motivations? One is that it seems to allow for a better account of what causes are and, in particular, what would have happened to them if a further relevant causal factor had come into play. For assuming (No Completeness), a cause is simply a plurality of relevant causal factors, A. Thus, in particular, whenever we consider a given cause and what would have happened if a further relevant causal factor B had obtained together with A, the crucial question **(Cause?)** “Would *the cause* still have obtained?” reduces to a question about the composition of A and B: **(Classical?)** “Would *A* have composed classically with B?” When discussing the holistic composition strategy, I was assuming that this picture was correct—I only argued that, on this picture, saving CN is hopeless. Now, I suggest that this very picture is incorrect. For it seems that, in the examples considered, the answer to (Cause?) is always negative: the cause would *not* have obtained if B had been there. This impression is incompatible with the picture on which the cause is A, and (Cause?) reduces to (Classical?): in at least some cases, the answer to (Classical?) is quite clearly positive—A and B would have composed classically. Yet, it perfectly fits the alternative picture on which the cause is A^+^—B is incompatible with the completeness fact in A^+^, so that the answer to (Cause?) is always negative, whatever the answer to (Classical?).

Consider the acceleration case: A is the relevant causal factors for the acceleration of an object *o*—say, the forces being exerted on *o*—and C is the fact that *o* accelerates accordingly. What would have happened if a further relevant causal factor B—a further force exerted on *o*—had obtained? Presumably, C would not have obtained. But it also seems that that the initial cause would no longer have obtained: the answer to (Cause?) is negative. For it does not sound right to say that the cause of C would still have been there, just as in the actual case, but that it would somehow have “failed” to cause C, or been “prevented” to do so; it sounds more natural to say that a new cause, including both A and B, would have been there instead—also succeeding in causing its own effect. Although the fact that an additional force is there seems to be irrelevant to whether the initial set of forces is still there—hence the positive answer to (Classical?) –, it *is* relevant to whether this set of forces still constitutes the *resultant* force, which in turn seems relevant to whether the actual cause still fully obtains—hence the negative answer to (Cause?). Those intuitions, which seem at least reasonable, are incompatible with the picture on which A is the cause and (Cause?) reduces to (Classical?). But they perfectly fit the alternative picture on which the cause is A^+^. What caused the actual acceleration of *o* was not simply the fact that there were certain forces exerted on it, but also the fact that there were no further forces.

Now take the room temperature example. How should we answer (Cause?)—would the *cause* still have obtained if B (the powerful cooler) had been there? Just as in the acceleration case, there seems to be something wrong in saying that the actual cause of C (the rise in temperature) would still have obtained, but would somehow have “failed” to cause C. Rather, there would have been a new cause, including A and B, which would have succeeded in causing its effect (a decrease in temperature). Thus, the answer to (Cause?) seems negative. On the old picture, the only way to account for this impression is to say that the answer to (Classical?) is also negative: A and B would have composed holistically. However, even assuming that they would have (which is in itself disputable), it cannot be the main reason for the impression that the initial cause would not have obtained anymore. For if it were, we would expect not to have that same impression when considering a clear(er) case of classical composition. Yet, in the acceleration case, there was the same impression. Whether composition would have been classical or holistic in the temperature case, what can clearly explain that same impression is that the actual cause is A^+^—namely A “alone”, in particular without B. The absence of B in the actual situation may or may not be relevant to the full obtaining of *the relevant causal factors, A*; but the absence of B definitely seems relevant to the full obtaining of *the cause* of C.

A second motivation for completeness is indirect: it concerns the relation between CN and the modally weaker thesis of causal *nomic* necessitarianism (CNN). As I have argued (Sects. 3.3–4), the other strategies to undermine the monocity argument, based on the rejection of (Classical Necessitation) or (Classical Composition), are hardly promising, so that the completeness strategy remains the only available one. As a result, (No Completeness) very plausibly stands or falls with the monotonicity argument against CN. But given the close links between CN and CNN (Sect. 3.1), it is easy to see that the same goes for the analogous monotonicity argument against CNN. Thus, no less than defenders of CN, defenders of the more general idea that causation supports (non-accidental) regularities have a good reason to go for completeness.

A different indirect motivation was already mentioned earlier: including a completeness fact in certain grounds is often considered as a legitimate move; and as those grounding cases look analogous to causal cases, it is unclear why including a completeness fact in causes should be less legitimate. Yet, this indirect motivation might be disputed. For it might be that the *specific reasons* why completeness is often considered as legitimate in the grounding case are not (clearly) applicable to the causal case, or anyway not relevant to the present defence of CN against the monotonicity objection. Indeed, one sort of argument for completeness in the grounding case simply relies on the very claim that GN is true, whether this claim is just assumed or independently motivated: GN is true; certain grounds (such as those considered earlier) are necessitating only if they include a completeness fact; therefore, they must include it. Such an argument will not be of much help here. First, although GN may just be assumed in certain contexts, as it is quite widely accepted, the same cannot be said about CN. And even when necessitation in the grounding case is not just assumed, but based on independent arguments, such arguments may not be as convincing once adapted to the causal case.^30^ Second, in the present context, we are anyway trying to undermine a particular objection, the monotonicity objection, against necessitarianism; thus, whether in the grounding or the causal case, it would be question begging to invoke necessitarianism itself—whether it is just assumed or independently motivated. Rather, what we need here is an argument for completeness that is *both* independent from necessitation and clearly applicable to the causal case as well.

One such argument concerns explanation. It is widely agreed that a (partial) ground provides a metaphysical explanation for what it grounds, with explanation understood in the objective, mind-independent sense—being a correct or appropriate metaphysical explanation does not depend on our interests or contingent capacities for understanding.^31^ And if partial grounds provide metaphysical explanations, it seems natural to think that grounds (i.e. full grounds) should provide *full*, or complete, metaphysical explanations. On that basis, one may argue that, in the relevant grounding cases, the ground should include a completeness fact. For instance, the mere fact that *a*, *b* and *c*, three mammals in Sarah’s garden, are hedgehogs—fact A—does not seem to provide a *full* metaphysical explanation for the fact that all the mammals in her garden are hedgehogs—fact C. By contrast, A *plus* the completeness fact that *a*, *b* and *c* are the only mammals in her garden—which excludes, for instance, that a fox is also there—seem to provide a full explanation for C. This reason for completeness is independent from necessitation, and arguably quite convincing.^32^ Importantly, it is also applicable to the causal case. For it is also widely agreed that causation is an explanatory relation, (partial) causes providing objective *causal* explanations. In particular, it is natural to think that causes (i.e. full causes) should provide *full* causal explanations. And this intuitively counts as a reason for completeness, just as in the grounding case. For instance, the fact that a number of forces are exerted on object *o* does not seem to provide a *full* causal explanation for its accelerating precisely as it does. Rather, it seems that a full explanation would also involve some completeness fact excluding, in particular, that other forces be exerted on it.

Note that a similar argument could be run, based on a different core feature that causation shares with grounding: both are relations of generation, or building.^33^ The argument would be distinct, but perfectly analogous to the one based on explanation: in short, just like the analogous sort of full grounds, full causes should be *fully* generative (i.e. they should fully generate, or bring about, their effects), which intuitively they would not be without including the relevant completeness fact—e.g. the fact that no further force is exerted on *o*.

More would be needed for a proper defence of completeness, but the above already gives us good motivations. There is, however, another issue to be addressed. For even granting that including *some appropriate* completeness fact in causes is both effective as a way to undermine the monotonicity objection, and legitimate on independent grounds, more needs to be said about what this completeness fact would look like. Indeed, this is one respect in which the causal and the grounding case may not seem to be on par: in the causal case, it may seem more difficult to find an acceptable formulation of the relevant completeness fact—one that is clearly able to exclude all alleged possible blockers while avoiding the sort of common drawbacks mentioned earlier (e.g. rendering necessitation vacuous). This is the problem to which I now turn.

### Formulating completeness: common difficulties

The problem of dealing with alleged blockers is not specific to the debate on CN—or CNN, or GN. It concerns various claims that a given relation “supports” some sort of modally loaded conditional. One is the claim that the relation between the triggering conditions and the manifestation of a disposition supports a counterfactual conditional: if x has disposition D (e.g. fragility), then, if D’s triggering conditions (x’s being struck) were to obtain, D’s manifestation (x’s breaking) would also obtain.^34^ Such claims, too, have been attacked on the basis of alleged potential blockers^35^ (e.g. a fragile object’s being protected by some packaging). In the case of causation, as in the other cases, a natural way to deal with blockers is to introduce some completeness fact or clause, T. And this may be done in two main ways. One is to include T *in* the cause itself (or e.g. in the disposition’s triggering conditions). The other is to add T as an *external* condition: if A causes C, then, *given T*, A necessitates C.^36^ In the context of our discussion, the former case amounts to rejecting (No Completeness) and including T in the cause itself, which is what I am suggesting here (the latter case would amount to renouncing necessitation *tout court*, as expressed e.g. by (Classical Necessitation), and defending only a weaker, conditional modal claim).

In the causal case, the main natural ways to formulate T face important difficulties (the case of dispositions is similar in this respect, while the grounding case is arguably less problematic). Those common ways may be divided into three categories, which I will now briefly present before considering them in turn. First, T may be an explicit *no-blocker* fact; the main problem, as I will argue, is that a cause’s necessitating its effect then becomes an entirely trivial matter. Second, T may be a totality fact that excludes everything but the relevant causal factors, A; the problem with the resulting “*all exclusive*” understanding of causes is that it is only applicable to “idealized” causes, not actual ones. Third, the cause may be taken to include everything in a certain space–time area (the “reverse light cone” of the effect), with T only excluding everything outside this area; the problem is that, on the resulting “*all inclusive*” understanding of causes, they include many facts that are intuitively irrelevant. Let us consider those three options in some more detail.

First, if T is meant to be an effective no-blocker fact, it should exclude, not only some explicit list of *particular* possible blockers, but *all* of them. The problem is that T will then run the risk of either being too vague or imprecise—“*ceteris paribus*”, “under normal conditions”, “excluding non-standard cases”—or being more precise but making the relevant conditional trivial, or vacuous.^37^ For instance, one may suggest that a cause, A^+^, has the form A *plus* T, where A is the relevant causal factors, and T is the (second-order) fact that, for any fact B, if it is possible that A and B obtain together without the effect C also obtaining, then B does not obtain. The explicit no-blocker fact, T, which directly refers to C and excludes the possibility of C’s non-obtaining if A obtains (together with any fact B), would indeed succeed in excluding all possible blockers; and it would be more precise than a general “in standard conditions” fact. However, it would also render the causal necessitation claim—A^+^ necessitates C—trivial. And applying this strategy to all causation, causes would become trivially necessitating. Indeed, on some historical accounts, it seems to be part of the very definition of a cause that it is necessitating.^38^ However, such moves would now appear to most of us as ad hoc attempts to save necessitarianism: intuitively, it is unclear why the very notion of cause should include necessitation.

And the point could be made even stronger. With causes of the form A *plus* T, it is not simply that causes are trivially necessitating: there is a sense in which they *logically* entail their effects.^39^ Here is briefly how the argument goes (I assume that “p” and “[p] obtains” are equivalent). Let us suppose that the cause obtains. Thus, A obtains, and so does T, namely the universally quantified fact [For any fact B, if it is possible that A and B obtain and the effect C does not, then B does not obtain], as well as any instance T_B_ = [If it is possible that A and B obtain and C does not, then B does not obtain], for any arbitrary fact B. In particular, there is a B that is a logical fact: B = [t], where t is some tautology. The corresponding instance, T_B_, is logically equivalent to [If B obtains, then necessarily, if A and B obtain, then C obtains]. And as B obtains as a matter of logic, by modus ponens, we get that [Necessarily, if A and B obtain, then C obtains] obtains; so does [Necessarily, if A obtains, then C obtains], since B is a logical fact; and so does [If A obtains, then C obtains], assuming that necessity is factive. As we were assuming that A obtains, by modus ponens, we get that C obtains. Thus, starting from the obtaining of the cause, we have logically derived the obtaining of the effect. Yet, even philosophers inclined to think that causes are necessitating, perhaps trivially so in some sense, would have been reluctant to claim that causation is indeed a *logical* relation.

The second main way to exclude blockers is with a totality fact making the cause “all exclusive”: roughly, the cause is A, namely the relevant causal factors, *plus* the fact that *that’s all*. The intuitive idea is to consider A as obtaining in isolation—just as some causal factors are put in quasi-isolation in scientific experiments, except that here isolation is total. The totality fact—*and that’s all*—may be expressed with T understood as a totality operator on facts.^40^ Roughly, T(A) is the (second-order) fact that no fact other than A obtains. Or, perhaps more simply, we may take T to be a factive operator, so that *A* becomes redundant in *A and T(A)*, and the cause is just T(A), amounting to A *plus* the fact that no other fact obtains—i.e. A *plus* [For any B, if B obtains, then B is identical to (included in) A]. The suggested formulation may of course be refined. In particular, T(A) excludes more than needed. For instance, as the idea is to have A in *causal* isolation, there may be no need to exclude “abstract” facts (facts involving only abstract entities), like [2 + 2 = 4]; we may not even need to exclude “concrete” facts outside the “reverse light cone” of s, where s is the spacetime location at which C obtains. We may then restrict totality to facts that have the property of being first-order, concrete facts in the reverse light cone of s—call that complex property “RLC(s)” (for “reverse light cone of s”). On the resulting view, the cause is the factive, restricted totality fact, T_RLC(s)_(A)—i.e. A *plus* [For any B, if B is an obtaining RLC(s), then B is (included in) A]. This formulation may still need further improvements.^41^ But it will do for our present purpose, which is to consider how the type of cause considered, consisting in A as being in isolation, is able to meet the challenges considered above. First, T_RLC(s)_(A) succeeds in excluding all alleged possible blockers, and the totality fact involved is arguably more precise than a general “in standard conditions” fact: it is a *ceteris absentibus* fact—all (concrete) things other than A being absent. Moreover, unlike the sort of formulation considered earlier, it does not render the necessitation claim vacuous or trivial: there is nothing in T_RLC(s)_(A) that already contains, or logically implies, C.^42^ However, this strategy has a major drawback: a fact like T_RLC(s)_(A) cannot be an actual cause, only an “idealization” of it—in actual cases, the relevant causal factors, A, do *not* obtain in isolation. Thus, we can only say that, *if* an ideal cause of form T_RLC(s)_(A) had obtained (in another possible world), then it would have necessitated its effect—at least, necessitation would not have been threatened by possible blockers. Yet, our main problem here is whether *actual* causes have possible blockers.

One natural way out is what I called the “all inclusive” option: the cause is not simply A in isolation, but *all* the first-order, concrete facts actually obtaining in the reverse light cone of s—all the actual RLC(s)’s—in isolation. Noting “I” (for “irrelevant”) the plurality of all the facts other than A that are also actual RLC(s)’s, the cause may be formulated as T_RLC(s)_(A, I)—i.e. A *plus* [For any B, if B is an obtaining RLC(s), then B is identical to (included in) A or I]. Unlike T_RLC(s)_(A), T_RLC(s)_(A, I) is now applicable to *actual* causes. And it still excludes all possible blockers, without making the necessitation claim trivial. However, first, it seems that a cause including both A and I, namely a whole reverse light cone of concrete facts, can only be the one instantiation of the corresponding repeatable *type* of cause—i.e. the only actual T_RLC(s)_(A, I)-like fact. Some may find it problematic, as we usually see particular causes as instantiations of repeatable, indeed often repeated, types.^43^ It is not obvious to me why this should count as an important problem: I can understand why, from a practical point of view, we may want types of causes to be relatively few, and often repeated; but it is unclear to me why its not being the case should be a problem in itself if what we are after is a correct metaphysical account of causation. Be that as it may, I think that the view under consideration faces a more important issue: what makes it difficult to accept a cause of the form T_RLC(s)_(A, I) as intuitive has less to do with I containing a huge number of facts than with its containing facts that, by hypothesis, are not relevant causal factors, and that seem, more generally, mostly irrelevant to C’s being caused.

Finally, there are general worries about causes including completeness facts, which apply to all types of formulation considered above: the “all inclusive” one, just considered; the “all exclusive” or “isolation” one, only applicable to idealized causes; and the no-blocker one, making causes trivially necessitating. For in all cases, the cause includes, or entails, a fact of the following form: [*For any fact B*, if B meets such-and-such conditions, then *B does not obtain*.] To take the simplest example, an unrestricted totality fact like T(A) includes [For any fact B, if B is not (included in) A, then B does not obtain]—and the same goes for the other formulations considered, except that the conditions on which B does not obtain are more complex. Such second-order facts, as part of the cause, may be objected on two grounds. First, they include *negative* facts, namely the *non*-obtaining of certain facts—indeed, an infinity of them. And one may argue that a cause should not involve anything negative: causation is a matter of generating, or bringing about, effects; but absences, it seems, are unable to bring about anything.^44^ Second, one may worry that the cause will involve an infinity of facts that seem intuitively irrelevant. As we have seen, T_RLC(s)_(A, I) already faces such a difficulty—I seems mostly irrelevant to C’s being caused. But the problem considered here is another, more general relevance problem, which also affects T(A) or T_RLC(s)_(A), for instance. For all those formulations involve a second-order fact that, *for any fact B*, if B meets certain conditions, then B does not obtain. And even forgetting about the “negativity” issue, the worry is anyway that all those B’s—including e.g. [2 + 2 = 4], [Today is Tuesday], [Socrates is a philosopher]–, and whether they meet the relevant conditions, may seem irrelevant to C’s being caused.^45^

### Formulating completeness: a suggested solution

The above briefly illustrated common (families of) ways of understanding causes as excluding alleged blockers. They all come with drawbacks, some of which are particularly important. Is there a better way? A natural idea, in the light of the previous general discussion about completeness (Sect. 5.1), is the following. It is assumed by both parties in the debate about the monotonicity objection that the cause includes A, namely the plurality A_1_, A_2_, A_3_,… of relevant causal factors (e.g. the causal factors relevant for the evolution of temperature in a given room). It is also agreed that these causal factors are the *only* (actual) relevant causal factors, so that together they form the *resultant* causal factor. Indeed, this latter fact is useful to explain why the effect obtains (e.g. why the temperature increases exactly as it actually does). However, the proponents of the monotonicity objection claim that it is not part of the cause; I now suggest that it is—the cause includes the completeness fact that A exhausts the relevant causal factors.

It should be stressed that what the relevant causal factors are, in each particular case, does not directly depend on C itself. As I already pointed out, what relevant causal factors are relevant to is not simply C, but a larger category or *domain* of potential effects, that I will call “D(C)”: when the effect C is a *particular* evolution of temperature in a given room, for instance, D(C) is the evolution of temperature in that room *in general*; when C is a *particular* acceleration of an object, D(C) is the movement or acceleration of that object *in general*. To put it otherwise, D(C) is related to C as is a determinable to one of its determinates, or a variable to one of its values. Thus, the relevant causal factors are, more explicitly, the *D(C)-relevant* causal factors.

Let me briefly address a potential worry.^46^ The above presupposes that there is a (non-arbitrary) way to associate any determinate effect C with a unique determinable D(C), which one might dispute—in particular, a determinate may in principle be a determinate of several determinables (e.g. scarlet is a determinate of both red and colourful). A helpful way to think of determinable-determinate relations is in terms of “determination spaces” (Funkhouser 2006, 2014, pp. 25–36). For instance, colourfulness has a three-dimensional determination space: being coloured is having three particular values falling within the ranges of possible values for the variables hue, saturation and brightness, respectively. Redness is a determinate of colourfulness because its determination space is a proper subset of the determination space for colourfulness: the space has the same three dimensions, but with more restricted ranges of possible values.

*Mutatis mutandis*, we may establish analogous determination relations between certain facts involving the instantiation of properties—relying on the determination relations between those properties. For instance, [Object *o* is red] is a determinate of the determinable [Object *o* is coloured]: the former’s determination space is a proper subset of the latter’s, which is constituted of all “point-like” facts of the form [Object *o* has values *h* for hue, *s* for saturation, *b* for brightness], for some triplet (*h*, *s*, *b*) in the three-dimensional determination space of colourfulness. In particular, it seems reasonable to think of any given effect C as involving the instantiation of some properties, and accordingly as being the determinate of some determinable D(C). For example, C = [*o* has acceleration (x’’, y’’, z’’) = (*1*, *0*, *-1*)], where 1, 0 and -1 are the particular values for the x-, y- and z-components of *o*’s acceleration in m/s^2^, is a determinate of determinable D(C) = [*o* has some acceleration], the latter’s determination space being constituted of all the facts [*o* has acceleration (x’’, y’’, z’’) = (*a*_*x*_, *a*_*y*_, *a*_*z*_)], for any (*a*_*x*_, *a*_*y*_, *a*_*z*_) in the three-dimensional determination space of all possible values for *o*’s acceleration.

Now, why think that D(C) is unique for any effect C? In the above example, another determinable for C would be D(C)* = [*o* has acceleration (x’’, y’’, z’’) ∈ [(0, 0, -2), (2, 0, 0)]], itself a determinate of D(C) = [*o* has some acceleration]. Yet, keeping in mind that here C is not any determinate fact but an effect, and that D(C) or D(C)* is not any determinable but what the relevant causal factors (e.g. the forces exerted on *o*) are supposed to be (objectively) causally relevant to, the two candidates are intuitively not on par. The property of *o* that the forces exerted on it are causally relevant to, the one that they play a causal role in determining within the relevant determination space, is *o*’s acceleration *tout court*, namely its property of having an acceleration—rather than its property of having an *acceleration-within-this-specific-range*. The latter property of *o* just does not seem sufficiently natural or joint-carving; neither does, in turn, the forces’s property of being causally relevant to that property of *o*.

The claim that any effect C comes with just one natural D(C) may require further argument, but the above suggests that it is at least reasonable. Let us now turn to a more precise formulation of the view of causes suggested above. For each particular effect C, the relevant causal factors are, more precisely, the *D(C)-relevant* causal factors—for short, the D(C)-RCF’s. The suggested cause, then, may be formulated as the following factive, restricted totality fact: T_D(C)-RCF_(A)—i.e. A *plus* [For any B, if B is an obtaining D(C)-RCF, then B is (included in) A]. Put simply, the cause amounts to *A and that’s all as regards relevant causal factors*.^47^

Let us now see how this way of understanding completeness compares to the main alternatives considered above (Sect. 5.2). First, the suggested completeness fact is arguably more precise than a mere “in standard conditions” fact, while still being general enough to exclude all possible blockers: if the cause is T_D(C)-RCF_(A), its obtaining is incompatible with the obtaining of any additional relevant causal factor—and thus, of any alleged blocker. Second, while excluding all possible blockers, it does not make the cause trivially necessitating, or CN vacuous: just like T_RLC(s)_(A, I)—the “all inclusive” cause—or T_RLC(s)_(A)—the “all exclusive” cause –, T_D(C)-RCF_(A) does not contain, or logically imply, C.^48^ Third, unlike the “all exclusive” view– i.e. T_RLC(s)_(A) as the cause –, the suggested view can apply to *actual* causes: unlike T_RLC(s)_(A), T_D(C)-RCF_(A) does not exclude *all* other facts (in the reverse light cone), but only further relevant causal factors—which by hypothesis do not actually obtain. Fourth, the proposed view is applicable to actual causes *without* their having to include a huge collection of irrelevant facts, I (all the concrete facts obtaining with A in the whole reverse light cone): unlike T_RLC(s)_(A, I)—the “all inclusive” cause –, T_D(C)-RCF_(A) is compatible with, but does not include, I.

Let us now briefly consider the more general worries about causes involving a fact of the form [*For any fact B*, if B meets such-and-such conditions, then *B does not obtain*]—in the case of T_D(C)-RCF_(A), the relevant fact would be [For any B, if B is a D(C)-RFC that is not (included in) A, then B does not obtain]. One worry was that such a fact includes negative facts—the non-obtaining of certain B’s; the other worry was that all those B’s (and whether they meet the given condition) are intuitively not relevant to C’s being caused. First, as those worries apply to all types of completeness facts, they are irrelevant to a comparison between the formulation suggested and the main alternatives considered. Second, it is unclear how much weight should be given to such worries. In particular, it is not obvious that a cause’s involving negative facts should in itself be problematic.^49^ Third, even assuming that those worries are serious, the general reasons for completeness presented in Sect. 5.1 may be considered as more important—so that T_D(C)-RCF_(A), as a candidate cause, may still be preferred to A, overall.

Fourth, it is not even clear that T_D(C)-RCF_(A) has to face the worries considered in the first place. For T_D(C)-RCF_(A), on current interpretation, amounts to A *plus* [The obtaining D(C)-RCF’s are at most A]. But we may instead understand the cause, T_D(C)-RCF_(A), simply as [The obtaining D(C)-RCF’s are *exactly* A]. *If* this fact is then taken to reduce to the conjunction of (a) [The obtaining D(C)-RCF’s are *at least* A] and (b) [The obtaining D(C)-RCF’s are *at most* A], then the worries under consideration may apply—at least if we take (b) to amount to [For any B, if B is a D(C)-RCF that is not (included in) A, then B does not obtain]. However, one may precisely resist the claim that the intended “exact fact”, T_D(C)-RCF_(A), reduces to an “at least fact”, (a), plus an “at most fact”, (b). Instead, T_D(C)-RCF_(A)—though necessarily obtaining just in case (a) and (b) obtain—may be considered as distinct from, and not reducible to, (a) plus (b).^50^ If so, then T_D(C)-RCF_(A) avoids the worries considered—it may still *imply* (b), but it does not *include* it. In general, it is common to take what I call “at least” and “at most” facts as the more basic building blocks in terms of which we may build “exact” facts. This may have various practical and other advantages. But I fail to see any clear *metaphysical* reason why an exact fact, such as [The people in the room are (exactly) Mary and Simon], could not also be considered as basic, at least not reducible to the relevant “at least” and “at most” facts.^51^

In sum, I suggest that a cause includes the fact that the actual relevant causal factors, A, exhaust the relevant causal factors. Beyond its precise formulation, which would probably need further refinements, the proposed way of understanding causal completeness avoids important difficulties faced by the main alternative ways considered earlier. More generally, the suggested completeness fact seems to be needed as part of the cause—not to make it necessitating, but to make it an intuitive full cause in the first place. If more or less relevant causal factors had obtained, not only would the effect have been different and incompatible with the actual effect, but the cause itself would have been different and incompatible with the actual cause. In order to get a precise cause, you need to specify what the relevant causal factors are *exactly*—not at least, or at most. In that respect, subtracting and adding causal factors are on par: just as the initial cause is lost if you *subtract* some of the relevant causal factors (which anyone would grant), it is also lost, and for analogous reasons, if you *add* some relevant causal factors. This seems to be a central feature of how causation works. It is the reason why, for instance, it is usually accepted that causal laws need to be formulated with some completeness (or *ceteris paribus*) clause, so as to determine what causal factors there are exactly. The proponents of the monotonicity objection would probably not deny that causation works in that way: that feature of causation is precisely what their objection mainly relies on, and what makes the possibility of blockers seem so plausible at first sight. All I am suggesting here is that we take that feature on board by acknowledging that completeness is a component of causes—not some external condition. What causes an effect is not simply the obtaining of certain relevant causal factors, but the fact that *exactly* those relevant causal factors obtain.

## Conclusion

Though quite common, and *prima facie* powerful, the monotonicity objection against CN can, and should, be resisted. Once carefully analysed, it relies on assumptions that ultimately threaten the weaker view of CNN, as well as GN—views that many, beyond friends of CN, may find plausible. To put it otherwise, the ultimate scope of the objection is, in two respects, wider than usually thought: it is not simply an objection against causal necessitation, but against (non-accidental) regularity, whatever the precise modal status; and it is not simply about causation, but plausibly a larger range of explanatory relations, including grounding. Beyond such general motivations to address the objection, each of the assumptions underlying it may be disputed on independent grounds. For instance, one may reasonably think that (Classical Necessitation) should be replaced by a stronger, non-monotonic notion of necessitation; and (Classical Composition) may reasonably be rejected on the grounds that some pluralities of causal factors may compose holistically.

The corresponding two strategies to defend CN, however, seem hopeless. The assumption which is crucial to reject, both to undermine the objection and for the general purpose of providing a satisfactory account of causes, is (No Completeness). My defence of the corresponding strategy included a proposed formulation of causal completeness that avoids common difficulties, as well as independent motivations for causal completeness in general—some of which relied on the suggested strong analogy between causation and grounding (indeed, given this analogy, some independent elements of my overall defence of CN may, in turn, bring further support to GN against the relevant monotonicity objection). Of course, even if this defence is convincing, it does not show that there are no good reasons to reject CN, or indeed the weaker, more widely acceptable thesis of CNN. But it does show that, if there are any such reasons, they are not to be found in common considerations regarding monotonicity and alleged blockers.

## Footnotes

1 See Aristotle, *Physics*, II.7.198b5–9; *Metaphysics* VI.3.1027a32–b6, IX.5.1048a5–15; see also Stein (2012). Other friends of CN (or some close variant) include Hobbes (1655, ch. 9); Spinoza (1677, Part I, Axiom III); Mill (1843, III, V, §3, p. 241); Harré and Madden (1975, e.g. pp. 5, 90–91); Ellis (2001); Heil (2012, ch. 6); Marmodoro (2015).

2 See Hume (1739, I, III, §IV–XIV).

3 Indeed, a variant of the monotonicity objection also threatens Hume’s (1739 I, III, §XIV) own regularist view of causation—see 3.1 below.

4 See, in particular, Russell (1913, p. 7), Schrenk (2010) and Mumford and Anjum (2011a, b, 2014).

5 For Mumford and Anjum (2011a, b, 2014), the main constituents of causes, namely what I refer to with the generic term “relevant causal factors”, are activated causal powers, understood as irreducibly dispositional. I remain neutral on that matter, as their monotonicity objection against CN does not depend on it.

6 See Mill (1843, III, V, §3, p. 239), Lewis (1973b, pp. 558–559), Mumford and Anjum (2011a, p. 32) and Heil (2012, pp. 126–130).

7 The view that the *relata* of causation are events is common [e.g. Davidson (1967), Lewis (1973b), Kim (1973)], but not unanimous: in particular, some indeed take them to be facts (Bennett 1988; Mellor 1995; see also Wilson 2018)—and there are still other candidates, e.g. states of affairs (Armstrong 1997).

8 See e.g. Rosen (2010), Audi (2012a); others prefer to formulate grounding as a sentential connective (*because*)—see e.g. Fine (2001, 2012b) and Correia (2010).

9 See e.g. Mumford and Anjum (2011b, p. 68; 2014, p. 105).

10 E.g. “dispositional essentialist” views, on which causal laws derive from the essential causal properties of the relevant natural kinds—see Shoemaker (1980), Swoyer (1982), Ellis and Lierse (1994), Ellis (2001), Bird (2007) and Heil (2012).

11 See e.g. Armstrong (1983) and Fine (2005).

12 See e.g. Hume (1739) and Lewis (1973a, §3.3).

13 See e.g. Russell (1913) and Mumford and Anjum (2011a, ch. 3, 2014).

14 See e.g. Fine (2001, 2012b), Correia (2005), Schaffer (2009); Rosen (2010), and Correia and Schnieder (2012); for some more sceptical views, see Sider (2012), Wilson (2014), Koslicki (2015) and Hofweber (2016).

15 See Correia (2005), deRosset (2010, 2013a, b), Rosen (2010), Audi (2012a, b), Fine (2012b, 2015), Trogdon (2013), Dasgupta (2014), Wilsch (2015). But there are also grounding contingentists—e.g. Dancy (2004, ch. 3), Schnieder (2006), Schaffer (2010), Chudnoff (2011, ms), Leuenberger (2014a) and Skiles (2015).

16 See e.g. Rosen (2010, p. 118), Fine (2012b, §1) and Wilson (2018, p. 731).

17 See Skiles (2015, §4.1).

18 On totality facts (in truthmaking), see Russell (1918, p. 207), Armstrong (1997, ch. 13), Heil (2003, pp. 70–71); on their use in grounding, see Rosen (2010, §8) and Fine (2015, §7). (See also §5 below.).

19 Leuenberger (2014a, pp. 157–161) offers a different, though analogous, example—roughly, A is some physical facts (without a totality fact), C is some phenomenal fact, A fully grounds C, but there is a possible blocker.

20 See e.g. Leuenberger (2014a, p. 152) and Skiles (2015, §4.1, §5.4, §6).

21 Indeed, if (Classical Composition), (No Completeness) and (Classical Necessitation) ultimately threaten GN, it seems that they should also threaten the weaker view that grounding involves non-accidental regularity. I argued for an analogous claim in the causal case (§3.1), and it seems that the argument may be adapted to the grounding case. If so, then keeping (Classical Composition), (No Completeness) and (Classical Necessitation) would be quite costly, not only for the defenders of GN, but indeed for all those taking grounding relations to support at least non-accidental generalizations [e.g. Rosen (2010, p. 131), Audi (2012b, p. 693), Schaffer (2016, p. 57) and Wilson (2018, p. 729)].

22 As opposed to e.g. necessitation *other things being absent*, or *interferers being absent*—such qualified notions have been suggested to capture, for instance, the modal relation between physical facts and other actual facts (Leuenberger 2008), or between a triggered disposition and its manifestation (Bird 2005).

23 Lowe (2012) aims at undermining Mumford and Anjum’s (2011a) monotonicity objection against CN; however, he does not reformulate the objection exactly as I do here. In particular, he takes them to hold, not exactly (MONOTONIC NECESSITATION), but the following claim: (P necessitates Q) entails that, for any R, (*If* P and R, *then* Q). To undermine this claim, he argues, not that *necessitation* is not monotonic—indeed he does not deny (Classical Necessitation) –, but that there are plausible formalisations of *conditionals* (“If…, then…”) that are non-monotonic, such as the following: If P, then Q *iff* $\square(P \rightarrow Q)\wedge (\lozenge P\vee\square Q)$. In the present context, however, one may indeed reject (Classical Necessitation) on the grounds that Lowe’s stronger, non-monotonic conditional is better at grasping the notion of necessitation, and then undermine (MONOTONIC NECESSITATION) on that basis (although I will argue shortly that such a strategy is not very promising).

24 For instance, one may argue that necessitation is better captured by the non-monotonic relation that Fine (2012a, pp. 235–236) calls “*exact* entailment”, or “*exact* consequence”—A exactly entails C *iff* any state that *exactly* verifies A (i.e., roughly, classically verifies A *and is wholly relevant to A*) also exactly verifies C.

25 The relevant debate opposed John Heil and Olivier Massin to Rani Anjum and Stephen Mumford (see Mumford and Anjum 2014, pp. 113–115).

26 The point that relevant causal factors may compose holistically was made by John Heil and Olivier Massin, as an answer to Mumford and Anjum’s monotonicity objection (Mumford and Anjum 2014, pp. 113–115).

27 When recognizing that composition may be holistic in some cases, Mumford and Anjum (2014, p. 114) stress that, in such cases, even if their “antecedent strengthening test” for causal necessitation is not failed, it is not passed either—it simply does not apply. Thus, what they seem to recognize is that their original (2011a) test—corresponding to the monotonicity argument formulated in §2.2—should be replaced with a modified test, corresponding to the *classical* monotonicity argument—on the new test, if a cause has possible *classical* blockers, then it is not necessitating.

28 Taking the composition of forces as a case of classical composition implies some form of realism about mere *component* forces—a view that is controversial (Cartwright 1983, p. 59; Wilson 2009), but plausible (Mumford and Anjum 2011a, pp. 38–44; Massin 2017). In any case, we may take a similar example that does not directly appeal to forces. Let A be the fact that two objects *o* and *p*, with respective masses *m*_*1*_ and *m*_*2*_, are at a distance *d* from each other; and suppose that A causes C, the corresponding acceleration of *o* and *p* towards each other. Clearly, there is a possible situation where A obtains together with B, the fact that a *further* object *q* (with mass m_3_ and a given position relative to *o* and *p*) is also around, and C does not obtain. And intuitively, A and B would have composed classically.

29 The linearity criterion for classical composition was suggested by Mumford and Anjum (2011a, pp. 86–87; 2014, p. 114)—more precisely, they suggest that non-linearity is one important reason why composition may be holistic.

30 One such argument for GN involves essence: roughly, when A grounds C, it is essential to A (or C, or both) that, if A obtains, then A grounds C (see Fine 2012b, p. 74). Assuming that grounding is factive and that essentiality entails necessity, GN follows. Another argument runs roughly as follows: if A grounds C, then A provides a good explanation for C; if A provides a good explanation for C, then A does not have “confounding cases”, namely possible cases where A obtains and C does not; thus, GN is true (see DeRosset 2010).

31 My use of “*providing* a metaphysical explanation” is meant to cover both *being* a metaphysical explanation (e.g. Fine 2001, p. 15; Litland 2013; Dasgupta 2014) and *backing* such an explanation (e.g. Rodriguez-Pereyra 2005; Audi 2012b, pp. 687–688; Schaffer 2012, 2016, pp. 58–59; Wilson 2018, pp. 728–729). For a more sceptical view on the systematic relation between grounding and explanation, see e.g. Wilson 2014.

32 It may be disputed, however: one may argue that the completeness fact is not explanatory relevant (Chudnoff ms); or that, even if it were, it would not automatically follow that it is part of the ground (Skiles 2015, §5.4).

33 See e.g. Skiles 2015, §1; Schaffer 2016, p. 50.

34 This is the left-to-right direction of the “simple conditional analysis” of dispositions (see e.g. Quine 1960, ch. 46).

35 Known as finks (Martin 1994), and maskers, or antidotes (Bird 1998).

36 For instance, Choi (2006) pursues the former strategy for dispositions; Bird (2005) pursues the latter strategy for dispositions and related laws of nature; Leuenberger (2008) pursues the latter strategy for physicalism as the claim that physical facts ground all actual facts.

37 See e.g. Martin (1994, pp. 5–6), Fara (2005) and Yli-Vakkuri (2010).

38 E.g. Hobbes (1655, ch. 9) and Mill (1843, III.V.3, p. 332).

39 I owe this further point to an anonymous referee for this journal.

40 On totality operators on facts, see Chalmers and Jackson (2001) and Leuenberger (2014b).

41 In particular, I am assuming that, in the definition of RLC(s), the reverse light cone of s is understood *strictly*—i.e. excluding s itself. This is appropriate assuming that all causation is non-simultaneous. In this case, as any possible blocker would have been an RLC(s), any possible blocker is excluded by T_RLC(s)_(A), as intended. However, at least some causes (or, more generally, relevant causal factors) might be simultaneous with their effects (Taylor 1966; Brand 1980; Mumford and Anjum 2011a, ch. 5). If so, then there may be possible *simultaneous* blockers, namely blockers that would have obtained at s; and those blockers, failing to be RLC(s)’s, are not excluded by T_RLC(s)_(A). Thus, to allow for simultaneous causation, we may rather understand the reverse light cone of s *broadly* (i.e. including s). The problem, then, is that T_RLC(s)_(A) even excludes C; and we certainly would like C to be compatible with the cause—without being already contained in, or logically implied by, it. Perhaps we may assume that a blocker B (e.g. a further force exerted on *o*) is never in itself *incompatible* with C (a given acceleration of *o*), whereas an effect other than C (a *distinct* acceleration of *o*) *is* incompatible with C. If this is correct, then a way to make T_RLC(s)_(A) compatible with both the effect obtaining and its failing to obtain, while still excluding all blockers, may be the following: T_RLC(s)_(A) is A plus [For any B, if B is an obtaining RLC(s), then B is identical to (included in) A *or B is identical to (included in) C or B is incompatible with C*].

42 In any possible world where T_RLC(s)_(A) obtains, it is excluded, in particular, that there is a blocker and C does not obtain; but nothing a priori excludes that there is no blocker and C fails to obtain for another reason: for instance, one may argue that the causal laws applying to the case in question are not deterministic, or not metaphysically necessary, in which case there clearly is a possible world where T_RLC(s)_(A) obtains but C does not.

43 See Russell (1913, pp. 8–9) and Mumford and Anjum (2011a, ch. 3).

44 See e.g. Mumford and Anjum (2011a, p. 66; and ch. 6).

45 For a similar worry about totality facts in grounds, see Skiles (2015, §4.1).

46 Thanks to an anonymous referee for pressing me on this point.

47 The formulation of T_D(C)-RCF_(A) may require some refinements. In particular, more than one plurality of facts may intuitively count as forming the resultant causal factor. In the temperature case, the relevant causal factors may be correctly identified with some macro-physical facts A_1_, A_2_, …(the fact that the heater is on, etc.), but they may also be correctly identified with micro-physical facts that ground them, respectively, A’_1_, A’_2_, … Likewise, in the acceleration case, one may say that the relevant causal factors are A_1_, A_2_, …, corresponding to the forces exerted on *o* (the fact that force f_1_ is exerted on *o*, etc.), but one may instead invoke facts that arguably ground A_1_, A_2_, …, namely facts A’_1_, A’_2_, … about the masses of *o* and other objects, the distances between them, etc. Thus, various pluralities of facts, at various “levels of fundamentality”, may correctly identify the relevant causal factors; and none of them should be excluded by the cause, T_D(C)-RCF_(A), where A is one such plurality. We may then say that T_D(C)-RCF_(A) is, more precisely, A plus [For any B, if B is a D(C)-RCF, then B is (included in) A *or B grounds A or B is grounded in A*]. Put simply, the cause amounts to A and that’s all as regards relevant causal factors *that neither ground nor are grounded in A*.

48 See fn 42 above.

49 See e.g. Mellor (1995, pp. 131–135).

50 This is an adapted version of Fine’s (2012b, §7) suggestion that we may take an unrestricted totality fact of the form T(a, b, c, …)—i.e. the fact that a, b, c, … are exactly the objects that there are—to be a basic fact that necessarily co-obtains, but is not identical, with the fact that a, b, c, … exist plus the universal generalisation that, for any x, x = a or x = b or … [For worries about such basic facts, see Skiles (2015, §4.2).]

51 A general worry about the proposed view may be that, once reformulated in terms of events (as many views of causation are), it would be committed to “totality events”—which are, in particular, a sort of second-order events. A detailed answer to that worry is beyond the scope of this paper, but here are two brief remarks. First, totality events, and second-order events in general, may not be very common, and I can see how they might be considered as somewhat unnatural, *prima facie*. Yet, it is still not obvious why they should be so problematic—in particular, substantially more so than the analogous sorts of facts. Second, in any case, there may be no need to reformulate the proposed view in terms of events. After all, thinking of causation in terms of facts, as I did in this paper, may be intuitive enough. Indeed, the view that the relata of causation are events is not unanimous, and some actually take them to be facts instead (see fn 7).
